# Regression dilution bias: Tools for correction methods and sample size calculation

**DOI:** 10.3109/03009734.2012.668143

**Published:** 2012-08

**Authors:** Lars Berglund

**Affiliations:** Uppsala Clinical Research Center (UCR), Uppsala University Hospital, Sweden

**Keywords:** Correction methods, measurement errors, regression dilution bias, SAS and R programs

## Abstract

**Background:**

Random errors in measurement of a risk factor will introduce downward bias of an estimated association to a disease or a disease marker. This phenomenon is called regression dilution bias. A bias correction may be made with data from a validity study or a reliability study.

**Aims and methods:**

In this article we give a non-technical description of designs of reliability studies with emphasis on selection of individuals for a repeated measurement, assumptions of measurement error models, and correction methods for the slope in a simple linear regression model where the dependent variable is a continuous variable. Also, we describe situations where correction for regression dilution bias is not appropriate.

**Results:**

The methods are illustrated with the association between insulin sensitivity measured with the euglycaemic insulin clamp technique and fasting insulin, where measurement of the latter variable carries noticeable random error. We provide software tools for estimation of a corrected slope in a simple linear regression model assuming data for a continuous dependent variable and a continuous risk factor from a main study and an additional measurement of the risk factor in a reliability study. Also, we supply programs for estimation of the number of individuals needed in the reliability study and for choice of its design.

**Conclusions:**

Our conclusion is that correction for regression dilution bias is seldom applied in epidemiological studies. This may cause important effects of risk factors with large measurement errors to be neglected.

## Background

In the last decades there has been a growing interest in the implication of random measurement variability, i.e. measurement errors, of biological variables (e.g. (1–5)). Repeated measurements on the same individual will vary around the usual value because of measurement error. Measurement error is defined as the deviation between an observed value and a usual value. The usual value can be conceived as an individual's long-term average value. The measurement error can be separated into technical error due to, for example, an imprecise measurement device (e.g. a food frequency questionnaire) and the individual's biological variation over time (e.g. seasonal variation of intake of vitamins). Technical measurement errors depend not only on the measurement device but also on errors when samples are drawn and transported and when results are reported. To reduce effects of biological variation measurements are sometimes standardized (e.g. measurement in fasting state). Some errors are systematic in nature, and calibration techniques may then be used for correction. However, correction of random errors of individual values cannot be done.

The size of measurement error can be assessed with a validation study, where observed values using an imprecise method are compared with observations from a gold-standard method without error (6), or with a reliability study, where observations are replicated with the same method.

**Figure 1.**
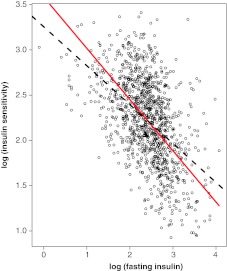
Dashed black line is ordinary regression line, and solid red line is regression line corrected for measurement error in measurement of fasting insulin.

One implication of measurement errors is that a bias is introduced into the estimation of coefficients when regression models or correlations are estimated (1). An example is the relation between insulin sensitivity measured with the euglycaemic insulin clamp technique (7) and fasting insulin (8). The latter is measured with noticeable error (9). If insulin sensitivity and fasting insulin are related to each other in a regression model where fasting insulin is measured once for each study participant the regression dilution bias will yield an underestimation of the risk for insulin resistance for a high long-term average of fasting insulin (Figure 1). This underestimation would be smaller with two or more measurements of fasting insulin for all participants and by use of the average of these values in the regression model. A more cost-efficient approach is to select a fraction of the participants for a replicate measurement of fasting insulin and to use the data from these participants to correct the regression coefficient for the measurement error in fasting insulin.

The rationale behind correction for regression dilution bias is that the usual level of a risk factor has an impact on disease progression. The measured values of the risk factor, being the usual levels with addition of random fluctuations unrelated to disease or disease progression, will consequently yield an underestimation of the risk factor's true impact (4,8,10,11).

The aims of this article were to give a non-technical description of designs of reliability studies, assumptions of measurement error models and the regression dilution bias and corrections methods for this bias in a simple linear regression model, and to provide software tools for corrected estimates and for sample size determination of reliability studies.

## Reliability studies

Reliability of a measurement method of a continuous variable is the similarity of repeated measurements administered on the same individual. The amount of measurement error is the variation seen over such repeated observations. When one sample from an individual is measured repeatedly or an individual is measured repeatedly with very short time intervals the variation is denoted technical measurement error. If an individual is measured repeatedly over two or more occasions (e.g. with intervals of 1 week or 1 month) the resulting variation is the total measurement error which is the sum of technical measurement error and biological variation. The present article is concerned with the total measurement error, and it is concentrated on the simple reliability design where a fraction of the participants in the main study is selected for a replicate measurement, e.g. a few weeks after the first measurement.

If it is not feasible to remeasure the risk factor on all participants in the main study, the style of selection of participants to a reliability study is important. The recommendation is usually to select a random sample from the main study (12). We have shown in two articles (8,11) that extreme selection, i.e. selection of the top- and bottom-ranked individuals for a second measurement of the risk factor, is more efficient than random sampling for estimation of a corrected regression coefficient. A reliability study design where the 20%–30% most extreme individuals in the main study are selected for a second measurement will often give almost the same corrected slope precision as remeasurements of all individuals in the main study.

## Regression models and measurement error models

The simplest regression model is where a continuous dependent variable is related to one continuous risk factor in a linear fashion. The slope in the model is the expected effect on the dependent variable of a one-unit increase of the risk factor.

The regression model is estimated without bias when the risk factor is measured without errors. When this is not the case, a model for measurement error structure must be assumed. The most common model is the classical measurement error model where a measurement error is added to the usual value. It is assumed that the measurement errors are unrelated to the usual values and to the measurement errors of the dependent variable. The classical model also assumes non-differential errors, i.e. the distribution of the observed risk factor conditional on the distribution of the usual risk factor gives no information on the distribution of the dependent variable.

## Regression dilution bias and correction methods for the slope in a simple linear regression model

The slope in the simple linear regression model is biased with a factor called the reliability ratio, which is defined as the variance of the usual values divided by the variance of the observed values of the risk factor. As the former variance is smaller than or equal to the latter the reliability ratio is between 0 and 1. It is well known (1) that the slope for the observed risk factor is the reliability ratio times the slope for the usual risk factor, and thus the ordinary estimator of the slope is biased towards zero. The term for the resulting underestimation is regression dilution bias (10).

When the reliability ratio is unknown it can be estimated from a reliability study. One estimator of the reliability ratio is the intra-class correlation coefficient ICC (12), which is estimated from a one-way analysis of variance model where individual is the factor. The slope in the linear regression of the second risk factor measurement on the first is another estimator of the reliability ratio.

The corrected estimator of the true slope is then the estimated observed slope divided by the estimated reliability ratio (1). Other estimators of the reliability ratio are described in the literature (12). The standard error of the estimated true slope is complex and depends on the reliability design, the precision of the estimated observed slope, and the precision of the reliability ratio (8).

However, the above-mentioned estimators do not utilize the covariance between the dependent variable and the second risk factor measurement. A maximum likelihood estimator incorporates this information into an estimator of the true slope (11). We provide program codes for this estimator and its standard error (see below). In a situation with a large amount of measurement error and a strong true relationship we showed that the standard error (and the width of a confidence interval) of the corrected slope was 54% lower with a maximum likelihood estimator and extreme selection compared to the above-mentioned estimators and random sampling (11).

## Application of methods for correction of regression dilution bias

There is an extensive statistical literature on regression dilution bias and methods for bias correction (e.g. (1,4,5)). Because most studies use only one measurement of each risk factor, systematic and substantial underestimations of the strengths of the real associations between risk factors and dependent variables will occur for risk factors with large intra-individual variability. As a consequence, in a situation with a positive association between a risk factor with measurement error and disease risk, high risk factor levels imply lower disease risk than in the case of no measurement error.

In multivariable models, with ranking of the importance of two or more risk factors with different degrees of intra-individual variability, it is essential to correct for the regression dilution bias in order to rank the per se effects of risk factors correctly.

Methods for bias correction are available in standard statistical packages. In spite of this a literature survey by Jurek et al. (13) reveals that out of 57 published articles in three leading epidemiological journals 39% do not mention regression dilution bias. None of the 57 articles corrects for this bias. The small number of practical applications of the methods for correction for regression dilution bias can be explained by limited training of biostatisticians and epidemiologists and of difficulties in funding two-stage designs with a main study and a reliability study or a validation study.

## When is it correct to correct?

There are circumstances when correction for regression dilution bias is not appropriate or unnecessary. When the aim of a study is to test the hypothesis of linear relation between two variables, but not to estimate the size of this relation, correction for regression dilution bias is not necessary. A test of the hypothesis the slope = 0 in the model with the observed risk factor is at the same time a test for the true slope = 0 as these parameters only differ by a factor. However, the power of the test decreases with increasing magnitude of measurement error.

If the reliability study is small relative to the main study its contribution to the length of the confidence interval for the corrected regression coefficient can be so large that the effort to collect replicates will not be worthwhile. In the example with insulin sensitivity as dependent variable and fasting insulin as risk factor the uncorrected estimated regression coefficient is –0.43 (95% confidence interval –0.47 to –0.39). If the fraction selected to the reliability study is 2% of the participants in the main study the corrected estimate is –0.57 (95% confidence interval –0.78 to –0.36). The example illustrates that, under certain circumstances, the corrected interval information will be that the upper limit is closer to zero compared with the uncorrected interval information.

The assumption of independence between measurement errors of the dependent variable and measurement errors of the risk factor is essential for the correction to be valid (14). A case in point is the relation between 24-h sodium excretion and blood pressure, where the former variable has large intra-individual variation. In the Intersalt study this relation is corrected for measurement error of 24-h sodium excretion (15). This correction is discussed by Smith and Phillips (16) who question if the assumption of unrelated measurement errors is justified.

Thus, each putative correction for regression dilution bias should consider if the study objective is hypothesis testing or estimation, the conceived length of the confidence interval for the corrected regression coefficient, and ascertainment of the assumptions of the measurement error model.

## Software tools

We provide programs in two commonly used statistical software packages, SAS and R, for maximum likelihood estimation of a corrected slope in a simple linear regression model assuming data for a continuous dependent variable and a continuous risk factor from a main study and an additional measurement of the risk factor in a reliability study when the reliability study is a fraction of the main study. Also, programs are given for estimation of the standard error of the corrected slope in a simple linear regression model given assumed values of the true correlation between the dependent variable and the risk factor, the reliability ratio, the number of observations in the main study and in the reliability study, and the design (random sampling or extreme selection) of the reliability study. The standard error may be used to decide the number of individuals needed in the reliability study and for choice of its design. Programs and their instructions for use can be found at www.ucr.uu.se/sv/downloads.

## Conclusion

We have highlighted the importance of considering measurement errors and their effects on risk assessments in simple linear regression models. Our conclusion is that correction for regression dilution bias is seldom applied in epidemiological studies. This may cause an important effect of a risk factor with large intra-individual variability to be neglected.

It is recommended to collect information about the amount of measurement error for the risk factor. Use of extreme selection for the reliability study in combination with a maximum likelihood estimator is a cost-efficient design for correction of regression dilution bias.
